# Good intentions, unintended outcomes: Impact of social assistance on tobacco consumption in Indonesia

**DOI:** 10.18332/tid/132966

**Published:** 2021-04-15

**Authors:** Teguh Dartanto, Faizal R. Moeis, Canyon K. Can, Suci P. Ratih, Renny Nurhasana, Aryana Satrya, Hasbullah Thabrany

**Affiliations:** 1 Department of Economics, Faculty of Economics and Business, Universitas Indonesia, Depok, Indonesia; 2 Institute for Economic and Social Research, Faculty of Economics and Business, Universitas Indonesia, Depok, Indonesia; 3 Department of Public Health Sciences, Faculty of Sport Science, Universitas Negeri Malang, Malang, Indonesia; 4 Urban Studies Program, School of Strategic and Global Studies, Universitas Indonesia, Central Jakarta, Indonesia; 5 Center for Social Security Studies, Universitas Indonesia, Central Jakarta, Indonesia; 6 Department of Management, Faculty of Economics and Business, Universitas Indonesia, Depok, Indonesia; 7 National Commission on Tobacco Control, Jakarta, Indonesia

**Keywords:** cigarette consumption, social assistance, socioeconomic indicators, impact evaluation, smoking intensity

## Abstract

**INTRODUCTION:**

Social assistance programs create an income effect that allows low-income groups to raise their consumption to improve their well-being. However, this may unintentionally induce an increase in their consumption of temptation goods, including tobacco. By analyzing five massive social assistance programs distributed by the government since 2007, we explore whether those programs may induce increased smoking intensity in Indonesia.

**METHODS:**

This study is a quantitative study that applies a Tobit regression, Difference-in-Differences (DiD) regression, Difference regression, and two-sample t-test, using the 2017 Susenas (National Socioeconomic Survey) and the 2007 and 2014 Indonesia Family Life Survey. Estimations using sociodemographic, regional, and social assistance dummy variables are used to explore the impact of the programs on the intensity of cigarette consumption in Indonesia, simultaneously assessing the relationship between cigarette consumption and socioeconomic conditions.

**RESULTS:**

Our estimations using Tobit regressions confirm that social assistance recipients consume 3.39 cigarettes per capita per week more than non-recipients. The DiD regressions on IFLS panel data show that social assistance programs significantly increase cigarette consumption by 2.8 cigarettes per capita per week. We also find that: 1) smokers have lower socioeconomic indicators than non-smokers in terms of nutrition and health and education expenditures, and 2) younger household members living with smokers have less educational attainment and higher average sick days.

**CONCLUSIONS:**

There is reasonable evidence to support the hypothesis that social assistance programs in Indonesia have contributed to the greater intensity of tobacco consumption among the recipients. The findings call for policy reforms in social assistance programs to be warier with the eligibility conditions for social assistance recipients. Adding new conditions related to smoking behaviors might reduce the smoking intensity of those in low-income groups and, in the long run, might improve the effectiveness of social assistance programs in raising the socioeconomic welfare of the low-income population.

## INTRODUCTION

Smoking has always been a forefront health issue in Indonesia, as the nation has one of the highest smoking prevalence rates in Asia^1,2^. The 2018 Riskesdas (*Riset Kesehatan Dasar*/Basic Health Survey) – a nation-wide survey – reports that 33.8% of the Indonesian population aged >15 years are active smokers. While this represents a decrease from 38.3% in 2013, the absolute number of smokers had no marked reduction^3^. Another worrying issue is that the nation’s youth smoking prevalence (aged 10–18 years) has increased from 7.2% in 2013 to 8.8% in 2016, and to 9.1% in 2018^3^. With the increase of active smokers, especially among the younger generation, policies to control smoking have been considered ineffective in lowering the number of smokers in Indonesia^4^. The persistent rise in prevalence may be due to cigarette prices being too cheap and affordable in Indonesia^5^. Complex cigarette tax systems also create opportunities for producers to avoid taxes, contributing to affordable cigarette prices^6^.

Examining the prevalence and intensity of smoking by expenditure groups allows a clearer depiction of those alarming conditions. Expenditure is chosen as it is a measure of consumption, which can reflect welfare more accurately in developing economies, and expenditure’s close correlation with income allows it to also be intertwined with social assistance recipiency^7^. While prevalence only measures the percentage of households who smoke, intensity measures how many cigarette sticks are consumed per week by households. The latter measure illustrates the severity of smoking behaviors, whereas the former only indicates their presence.

**Figure 1:**
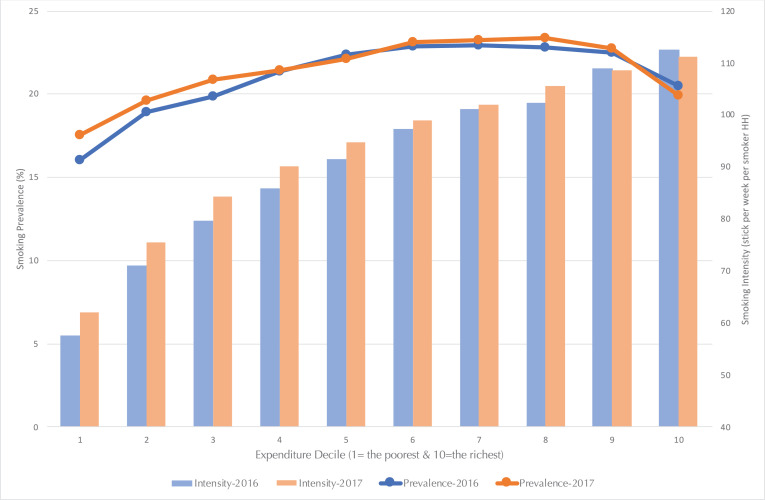
Smoking prevalence per expenditure decile

Figure 1 shows that from 2016 to 2017, both the prevalence and intensity of smoking among the low-expenditure population have increased faster than for those in the higher deciles. This condition is similar to that in the US, where low-income groups have a higher smoking prevalence than high-income groups^8^. The figures in Indonesia are even more disquieting as the rise in the smoking prevalence among the lowest first three expenditure deciles are 1.47%, 0.66%, and 1.04%, respectively, whereas the highest three deciles only experienced a smoking prevalence rise of 0.59%, 0.24%, and -0.57%, respectively. Moreover, the smoking intensity among smokers in the lowest first four deciles increased by 8%, 6%, 6% and 5%, respectively, while the highest four deciles have increased by only 1%, 3%, 0% and -1%, respectively. Thus, it is imperative to ask how groups with lower ability to spend are able to fund their increased smoking consumption, and why the significant increase in prevalence and intensity occurred largely in the first three deciles.

Assuming cigarettes are normal goods, the increase in prevalence as well as intensity must be driven by cigarette prices and income. However, as yearly hikes in excise taxes have raised cigarette prices and poor households are more sensitive to those price increases^4^, the notable increase in smoking intensity among the poor must be driven by an increase in their income during the 2016–2017 period. A possible extra source of income, which expands the consumption possibility for the poor, is the massive social assistance distributed in those years as part of the Indonesian government’s commitment to distribute assistance to those within poor and vulnerable income groups (the bottom 40% of the population) in order to foster an increase in their welfare and help lift low-income families out of the cycle of poverty through investments in human capital (e.g. education and health)^9^.

By 2017, 25 social assistance programs (including energy subsidies) that cost around IDR 204 trillion (US$14.6 billion) were distributed to almost 100 million people in poor and vulnerable groups^10,11^. On average, a household is able to raise its monthly expenditure by 7.64% through the three major social assistance programs *Program Indonesia Pintar* (PIP, or Indonesia Smart Card/Scholarship), *Program Keluarga Harapan* (PKH, or Conditional Cash Transfers) and *Kartu Keluarga Sehat* (KKS, or Family Welfare Card/Unconditional Cash Transfers). The 7.64% increase is equivalent to about IDR 45000 per month^12^, and is consistent with multiple studies that have demonstrated the effectiveness of social assistance or social safety in reducing poverty, with particular success in alleviating chronic poverty^13-15^.

The concern lies in that the additional resources received through social assistance programs may induce a result similar to that of an income effect, with the additional resources being used for non-essential goods such as tobacco and alcohol or with the additional resources allowing households to allocate more of their income towards non-essential goods (as the social assistance pays off previously burdensome food, education and medical bills). Such a concern has been raised in Zambia and Malawi by the government and aid agencies that believe that when men control the cash provided, they more frequently spend it on alcohol and cigarettes, rather than on food or basic necessities^16^. If this hypothesis is true, then the goal of social assistance to increase the welfare of its recipients will have the opposite effect; it is critical to understand whether or not social assistance recipients correctly use the aid that is given.

However, past research has not found that households were using government cash transfers to purchase tobacco and alcohol^17^. In Indonesia, ambiguous results were found, with unconditional cash transfers moderately reducing the demand for tobacco and alcohol during the first disbursement, but increasing demand for those temptation goods during the second disbursement^18^. Recent research has shown no significant evidence that unconditional cash transfers drive risky behaviors such as smoking^19^. Thus, the impact of social assistance on smoking behaviors is still inconclusive and questionable. Yet, social assistance may not have a strong enough effect to encourage smoking among non-smokers, but it may be sufficient to increase smoking intensity among smokers. Thus, this study aims to provide new insights into how social assistance might influence the intensity rather than the prevalence or intention to smoke, and how the intensity of smoking affects socioeconomic characteristics among households and youths.

### Social assistance programs in Indonesia

The years 2012 to 2017 saw a massive shift in the government budget away from fuel subsidies to spending on electricity subsidies and targeted social assistance programs, distributed to eligible households based on the unified database managed by the Ministry of Social Affairs, containing detailed socioeconomic information for almost 28.8 million households in 2018^10^. Aside from electricity and Liquefied Petroleum Gas (LPG) subsidies, the five main social assistance programs in Indonesia in 2017 were PIP, KIS (Indonesia Health Card, or Kartu Indonesia Sehat), PKH, KKS, and Rastra which was later renamed as BPNT (*Bantuan Pangan Non Tunai* or Basic Food Voucher). Through the unified database, the government has tried to integrate a conditional cash transfer program with the PIP and KIS programs, marking one of the government’s strides towards a more comprehensive, interlinked social assistance approach^12^. In total, government spending on household social assistance programs has risen more than fivefold between 2007 and 2016, from IDR 14.2 trillion to 78.3 trillion^12^.

**Figure 2:**
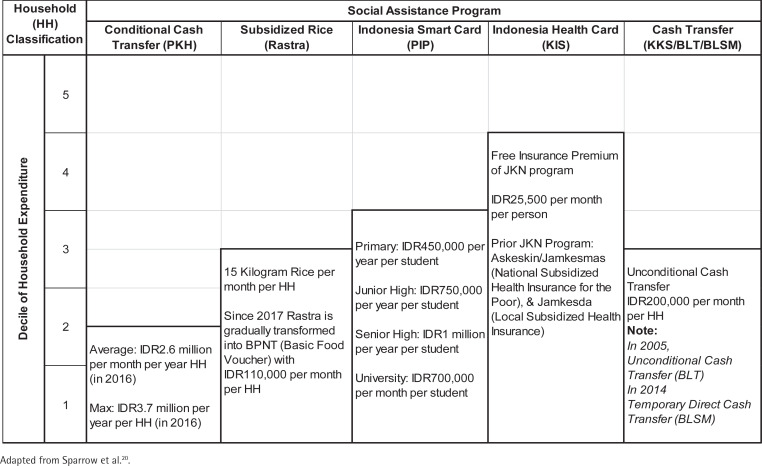
The main social assistance in Indonesia during 2007–2017

This study assesses the impacts of five social assistance programs with the broadest recipients: PKH, Rastra, PIP, KIS and KKS. The coverage and benefits of these programs are shown in Figure 2 ^20^. These programs are given to the poorest 15% to 40% of households, most of them regardless of gender and age. In particular, the PKH program, which was established in 2007 to reduce long-term poverty by increasing access to basic needs such as health services, education, and nutrition, saw rapid growth in its coverage. Only 387947 of Indonesia’s poorest households were covered by the program in its inaugural year; as of 2017, more than 6.23 million households received its benefits, with the program’s budget rising more than nine-fold. The PKH specifically targets households with pregnant women, school-age students, members with disabilities, children aged <5 years, and the elderly. Beneficiaries are able to receive conditional cash transfers for up to nine years. In 2016, the maximum amount in transfers that could possibly be received by a household was raised to IDR 3.7 million a year.

Rastra was initially intended to assist the poor’s ability to afford food in the wake of the 1997/98 crisis, but has since been expanded due to the large proportion that food takes up in a poor household’s expenditures. The World Bank estimates that two-thirds to three-quarters of the Indonesian poor’s expenses are spent on food, thus making the Rastra critical in alleviating poverty and ensuring a stable food supply for households amidst price volatilities^12^.

The PIP aims to tackle high enrolment costs, which present a large hurdle that prevents children from receiving education. The 18.2 million students covered by the PIP in 2016 is over four times the 4.6 million students covered by the program when it first began in 2008, and its budget has risen from IDR 1.2 trillion to IDR 11.3 trillion^10^. Households receive between IDR 0.45 million to IDR 1 million per year, depending on the child’s level of education (Figure 2). Poor families in the bottom 25% of incomes with school-age children, aged 6–21 years, are eligible to receive the PIP. In terms of health, all government social insurance schemes were integrated into the JKN program in 2014, with a free premium (KIS) for poor and near-poor households. The KIS program takes the largest share of the government’s social insurance budget at almost 40%, with IDR 21.1 trillion spent in 2017 for 96 million people, compared to IDR 4.6 trillion in 2007^10,12^.

Lastly, the KKS aims to put various social assistance programs under one umbrella, so that households in possession of the KKS can automatically be eligible for various social assistance programs including the PKH, Rastra, PIP and KIS. Households with KKS receive cash transfers of IDR 0.2 million per month per household (Figure 2). In 2017, the government distributed around IDR 12.7 trillion to 6.2 million KKS recipients^10^. Thus, although the eligibility conditions for the five programs generally overlap, few households receive the full benefits of all five. Regardless, our earlier calculations, which reveal a 7.64% difference in monthly expenditures between recipients of the PIP, PKH, KKS and those who are not recipients, show that the programs have a marked impact on a household’s ability to consume more^19^.

However, despite their marked impact, common problems of social assistance targeting still persist, with the most common being errors of inclusion (those who are not eligible, i.e. the non-poor receiving social assistance) and errors of exclusion (those who are eligible, i.e. the poor not receiving social assistance). Indonesia has made significant strides to reduce these errors, but they remain pervasive^21^ . For instance, exclusion errors result in only 47% of eligible households in the lowest consumption decile receiving PKH assistance. Meanwhile, inclusion errors mean that nearly 20% of households in the sixth consumption decile receive PKH assistance despite being ineligible for it. Inclusion errors are of greater concern in our study as they may encourage consumption of temptation goods including cigarettes because the error results in households receiving additional resources when they do not really need social assistance.

## METHODS

### Data

The data used in this research are from the Indonesia National Social Economic Survey (Susenas) and the Indonesia Family Life Survey (IFLS). The Susenas is a socioeconomic survey of almost 0.3 million households conducted twice a year (March and August) by Statistics Indonesia or Badan Pusat Statistik (BPS). We use mainly the 2017 Susenas data (see Supplementary file Table S1 for descriptive statistics), with a focus on social assistance variables and five social assistance programs: PKH, Rastra, PIP, KKS and KIS. For robustness and consistency checks, we also use the 2016 Susenas to confirm our results.

The IFLS is a socioeconomic survey with data collection carried out in 5 waves (1993, 1997, 2000, 2007, and 2014, respectively) by the RAND Corporation. The descriptive statistics of the IFLS data are shown in Supplementary file Table S2. The respondents from the 1993 survey are re-surveyed in the following waves with low levels of attrition; therefore, the data can be used for panel (cohort) analysis^22^. The survey is representative of 83% of the population in Indonesia, and is conducted in 13 provinces. Although the distribution of social assistance programs in the period 2007–2014 was not as widespread as in the years after, the panel data of the 4th and 5th IFLS waves are the best available data for conducting impact evaluation and are thus used for this study’s purposes.

### Empirical strategy: Tobit model, DiD regression, Difference regression, and t-test

This study employs three empirical strategies sequentially. First, we estimate the relationship between social assistance and smoking behaviors using a cross-sectional approach to establish an initial association between the two variables. Then, using a panel impact evaluation method, we establish the impact of social assistance on smoking behaviors – in particular, on smoking intensity. Finally, we compare socioeconomic indicators between social assistance recipients who are smokers and non-smokers to understand the adverse differences in welfare outcomes between the two groups. While the cross-sectional approach can reveal relationships of correlation, the panel impact evaluation method adds strength to the relationships found as it allows for inferences of causal relationships. After identifying how social assistance can impact tobacco consumption, the comparison of socioeconomic indicators completes the analysis by identifying how tobacco consumption can in turn affect the socioeconomic outcomes which are the outcomes that social assistance programs aim to improve. This is critical because if social assistance programs inadvertently result in higher tobacco consumption, and if households with higher tobacco consumption possess worse socioeconomic indicators, then the relationship between social assistance programs and tobacco consumption poses the risk of compromising the effectiveness of the programs.

### Association of social assistance and smoking intensity: Cross-sectional analysis

We evaluate the relationship between the distribution of social assistance and cigarette consumption using the Susenas dataset at household level. The Tobit regression is used to analyze this phenomenon because the respondents who do not have smoking expenditures will have their smoking expenditures and quantity of cigarette consumption censored at zero. This will cause estimators to be biased if we conduct an ordinary least squares (OLS) analysis. Thus, the Tobit regression is applied, with the dependent variable being the per capita consumption of cigarette sticks. We also control sociodemographic variables, including, urban/rural, average household years of schooling, and electricity access. Whereas regional variables are island variables (Sumatra, Nusa Tenggara, Kalimantan, Sulawesi, and Maluku-Papua, with Java as the base) to capture regional/cultural differences between islands. Although this approach is unable to clearly show a causal relationship between social assistance and cigarette consumption, applying a Tobit regression on the Susenas dataset across all provinces provides a comprehensive initial picture regarding the relationship at the national level. The model is as follows (Equation 1):

Cigaretteconsi=γ1SocialAssisti+∑j=1JθjΔSocioDemoji+∑l=1LγlRegionalli+ui

### Impact evaluation of social assistance on smoking intensity: A panel data analysis

We estimate more accurately the impact of social assistance on cigarette consumption by employing the impact evaluation method of Difference-in-Differences (DiD) following the method used to calculate the impact of Indonesia’s subsidized national health insurance (*Asuransi Kesehatan Untuk Keluarga Miskin,* Askeskin) on multiple health utilization indicators in the 2005–2006 Panel Susenas data^23^. The DiD method conducted is the standard DiD (Equation 2, shown below) and with a Difference regression that excludes time invariant variables (Equation 3)^24,25^. The standard DiD uses the fixed effect and Tobit random effect regression with the variables of social assistance, a dummy to indicate the IFLS year that captures the different outcomes across time, and the interaction between the two variables. The interaction term will indicate whether the cigarette consumption of recipients of social assistance grows faster than that of non-recipients. We included the household fixed effect to control time-invariant unobserved heterogeneity of households.

The Difference regression uses the difference in the cigarette consumption between 2014 and 2007 as the dependent variable and uses as independent variable the difference between a person’s social assistance recipient status in 2014 and 2007. The number of observations in the Difference regression will be half the number of observations from the standard DiD as the differencing of variables causes individuals to be only counted once.

The IFLS panel data enable a clear division between the control and intervention groups that allows us to explore whether households receiving social assistance during this period also experience an increase in their cigarette consumption. Therefore, the estimation results regarding how social assistance (the treatment) affects smoking behavior are stronger because they compare recipients with their counterfactuals, isolating the social assistance effect towards smoking behaviors^24,25^. The most critical concern in DiD is the parallel-trend assumption that means unobserved characteristics affecting program participation do not vary over treatment with the treatment status^24^.

The social assistance variables used are those for PKH, Rastra, BLT (former KKS), Askeskin (former KIS) and a variable to indicate recipience of at least one kind of social assistance (BANSOS). The social assistance variables capture whether individuals received the social assistance in year t. However, the outcome of KIS’s intervention does not satisfy the parallel trend assumption (Supplementary file Table S2). Therefore, we excluded Askeskin/KIS for the DiD estimations. The models for the standard DiD and Difference regression are, respectively, as follows:

Cigaretteconsit=θ1SocialAssistit+θ2Yearit+θ3SocialAssistit×Yearit+∑j=1JθjSocioDemojit+∑l=1LθlRegionallit+εit

and

ΔCigaretteConsi=θ1ΔSocialAssisti+∑j=1JθjΔSocioDemoji+∑l=1LθlΔRegionalli+ui

where *CigaretteCons* is cigarette consumption measured as sticks per capita per day, *SocialAssist* is the dummy for the four aforementioned kinds of social assistance variables received by households (recipient of social assistance=1, non-recipient of social assistance=0), Year is the dummy variable for time (2014=1; 2007=0), *SocioDemo* is the household fixed effect that includes a vector of sociodemographic variables including years of schooling, urban–rural location, the quintile rank of households, and access to electricity, *Regional* is the dummy variable to indicate whether the household is located in Java or outside Java; *∆* represents the change during 2007–2014, *i* represents household, *t* is the time period, and *ε* and *u* are the error terms.

### Socioeconomic indicator differences between smokers and non-smokers: t-test analysis

Lastly, we estimate the difference in the socioeconomic outcomes between the recipients of social assistance who are smokers and recipients who are non-smokers. Finding a significant difference would lead to the conclusion that smoking behaviors reduce the effectiveness of social assistance programs in achieving welfare improvement. We use the t-test for mean differences on the socioeconomic indicators of smokers and non-smokers. The formula for the t-test is the following:

$$t=\frac{\left({\bar{X}}_{1}-{\bar{X}}_{2})-(\mu_{1}-\mu_{2}\right)}{\sqrt{\frac{(n_{1}-1)s_{1}^{2}+(n_{2}-1)s_{2}^{2}}{\left(n_{1}+n_{2}-2\right)}\times }\sqrt{\frac{1}{n_{1}}+\frac{1}{n_{2}}}}$$

where *X* is the sample average of *X*, *n* is the number of observations in each group’s sample, *s^2^* is each group’s sample variance, *μ* is the population average, and 1 and 2 refer to group-1 and group-2. We often assume that (*μ^1^-μ^2^* ) equals zero. The socioeconomic indicators that we evaluate using the t-test are consumption per capita per month (including food consumption of calories, proteins, fats and carbohydrates), health indicators (average days of sickness and inpatient treatment, average days of sickness for household members aged <15 years, health expenditures per capita), education indicators (years of schooling of household members aged <15 years, drop-out rates of household members aged <15 years, education expenditures per capita). The analysis utilizes the Susenas data.

## RESULTS

### Social assistance and cigarette consumption: Cross-sectional approach

Tobit estimations demonstrate that receiving social assistance is indeed positively correlated with cigarette consumption per capita. Table 1 shows significant positive association between receiving social assistance (consistently for each kind of social assistance) and cigarette per capita per week consumption. This means that households receiving social assistance tend to have higher cigarette per capita per week consumption. The program most highly associated with smoking consumption is the Rastra, where a household receiving Rastra will consume 4.52 cigarettes per capita per week more compared to households that do not. This is followed by PKH (3.51), KKS (2.89), PIP (2.55), and KIS (0.78). If the household receives at least one social assistance program, it will consume 3.39 cigarettes per capita per week more than non-receivers of social assistance. We also estimate the Tobit regressions using the 2016 Susenas and find consistent results (Supplementary file Table S3).

**Table 1:** Tobit regression of cigarette consumption in 2017 (stick per capita per week)

No.	Variables	Tobit regression Cigarettes per capita (standard error)					
1	Recipient of Rastra (1=recipient; 0=non-recipient)	4.523*** (0.009)					
2	Recipient of PIP (1=recipient; 0=non-recipient)		2.548*** (0.014)				
3	Recipient of KKS (1=recipient; 0=non-recipient)			2.885*** (0.012)			
4	Recipient of PKH (1=recipient; 0=non-recipient)				3.507*** (0.017)		
5	Recipient of KIS (1=recipient; 0=non-recipient)					0.777*** (0.008)	
6	Recipient of at least one social protection (1=recipient; 0=non-recipient)						3.391*** (0.009)
7	Urban (1=urban; 0=rural)	-4.033*** (0.009)	-4.684*** (0.009)	-4.626*** (0.009)	-4.677*** (0.009)	-4.701*** (0.009)	-4.306*** (0.009)
8	Average household member years of schooling	-0.559*** (0.002)	-0.658*** (0.002)	-0.630*** (0.002)	-0.650*** (0.002)	-0.651*** (0.002)	-0.592*** (0.002)
9	2nd expenditure quintile (1=2nd quintile; 0=others)	8.822*** (0.013)	8.688*** (0.013)	8.779*** (0.013)	8.776*** (0.013)	8.635*** (0.013)	8.768*** (0.013)
10	3rd expenditure quintile (1=3rd quintile; 0=others)	13.49*** (0.013)	13.13*** (0.013)	13.24*** (0.013)	13.21*** (0.013)	13.00*** (0.013)	13.32*** (0.013)
11	4th expenditure quintile (1=4th quintile; 0=others)	17.00*** (0.013)	16.37*** (0.013)	16.52*** (0.013)	16.44*** (0.013)	16.18*** (0.013)	16.75*** (0.013)
12	5th expenditure quintile (1=5th quintile; 0=others)	19.46*** (0.015)	18.41*** (0.015)	18.54*** (0.015)	18.43*** (0.015)	18.19*** (0.015)	19.17*** (0.015)
13	Living in Sumatera (1=Sumatera; 0=others)	6.026*** (0.010)	5.603*** (0.010)	5.677*** (0.010)	5.630*** (0.010)	5.575*** (0.010)	5.699*** (0.010)
14	Living in Nusa Tenggara (1=Nusa Tenggara; 0=others)	-5.826*** (0.019)	-6.415*** (0.019)	-6.389*** (0.019)	-6.394*** (0.019)	-6.345*** (0.019)	-6.221*** (0.019)
15	Living in Kalimantan (1=Kalimantan; 0=others)	2.930*** (0.018)	2.066*** (0.017)	2.165*** (0.017)	2.076*** (0.017)	2.050*** (0.017)	2.549*** (0.017)
16	Living in Sulawesi (1=Sulawesi; 0=others)	3.660*** (0.016)	3.093*** (0.016)	3.099*** (0.016)	3.151*** (0.016)	3.082*** (0.016)	3.206*** (0.016)
17	Living in Maluku-Papua (1=Maluku-Papua; 0=others)	-5.516*** (0.028)	-5.759*** (0.028)	-5.712*** (0.028)	-5.603*** (0.028)	-5.883*** (0.028)	-6.047*** (0.028)
18	Electricity (1=have electricity; 0=others)	1.137*** (0.033)	1.290*** (0.033)	1.457*** (0.033)	1.417*** (0.033)	1.365*** (0.033)	1.364*** (0.033)
	Constant	-1.213*** (0.034)	1.721*** (0.034)	1.041*** (0.034)	1.506*** (0.034)	1.664*** (0.034)	-1.154*** (0.035)
	Observations	67487588	67487588	67487588	67487588	67487588	67487588

*** p<0.01, **p<0.05, *p<0.1.

The control variables present several notable results. Demographic variables play crucial roles, with households living in urban areas generally having lower smoking expenditures than those of their rural counterparts. An increase in the average education of the household will decrease per capita per week consumption of smoking products, while households with electricity access will have higher cigarette consumption. Socioeconomic factors such as expenditure show that higher expenditure quintiles are largely associated with higher cigarette consumption. Finally, the regional variables reveal that compared to households in Java, households in Nusa Tenggara and Maluku-Papua tend to have lower cigarette consumption. Meanwhile, households in Sumatera, Kalimantan, and Sulawesi tend to have higher cigarette consumption than those in Java.

These results imply that cigarettes are a normal good, where cigarette consumption increases together with income. Social assistance either in the form of cash transfers (PIP, KKS and PKH) or in-kind transfers (Rastra, KIS) theoretically increase the household incomes of recipients. Recipients of in-kind social assistance, such as Rastra and KIS, can reallocate their expenses for rice and health to other needs or products, including cigarettes. However, unlike KIS that only indirectly increases household income, the social assistance programs of PIP, KKS, PKH and Rastra are directly transferred to households’ bank accounts, therefore directly increasing the incomes of households in a notable manner. This may contribute to the low effect of KIS on the consumption of cigarettes.

### Social assistance and cigarette consumption: DiD regression and difference regression approaches

The results of the regression in Table 1, using a representative sample from the entirety of Indonesia, illustrate an initial indication that social assistance can increase cigarette consumption; however, we cannot strongly conclude any causalities between them due to several endogeneity issues. Using the standard impact evaluation method of the DiD, it is explored further whether a causal relationship exists between receiving social assistance and intensity of smoking behaviors. Our DiD regression using fixed effects and the Tobit regression infer that individuals who received the social assistance of Rastra, BLT, and PKH, and those who at least received one (BANSOS) program, tend to have higher cigarette consumption growths than those of individuals who did not receive social assistance (Table 2). When households received the Rastra in 2014 but did not receive it in 2007, their cigarette consumption increased by 0.381 cigarette sticks per day or 2.67 more cigarette sticks per week than those that did not receive Rastra (variable *SocialPro*×*Year*). Moreover, those who received cash transfers (BLT/ BLSM) tend to increase their cigarette consumption by 0.258 cigarettes per day (or 1.81 cigarettes per week) more than those without the transfers. Meanwhile, individuals receiving PKH had no significant difference in their cigarette consumption growth compared to non-recipients. These results have been controlled with consumption, electricity access, urban/rural household location, Java/Non-Java household location, education, and age of the individual.

**Table 2:** Estimations of the impact of social assistance on cigarette consumption 2007–2014: DID regression

*No.*	*Variables*	*PKH*		*RASTRA*		*BLT/BLSM*		*Received at least one BANSOS*	
		*Fixed*	*Tobit*	*Fixed*	*Tobit*	*Fixed*	*Tobit*	*Fixed*	*Tobit*
1	SocialPro×Year	0.116 (0.925)	1.68 (2.542)	0.381*** (0.089)	0.819*** (0.236)	0.258** (0.105)	0.475* (0.272)	0.398*** (0.093)	0.710*** (0.246)
2	Social protection (1=recipient; 0=non-recipient)	0.286 (0.916)	-0.339 (2.517)	-0.271*** (0.086)	-0.0441 (0.211)	-0.089 (0.096)	0.416* (0.232)	-0.303*** (0.085)	-0.066 (0.211)
3	Year (1=2014; 0=2007)	0.476* (0.283)	0.285** (0.141)	0.271 (0.288)	-0.144 (0.191)	0.408 (0.285)	0.185 (0.159)	0.244 (0.290)	-0.163 (0.213)
4	Per capita expenditure (million IDR)^a^	0.438*** (0.042)	0.967*** (0.101)	0.456*** (0.043)	1.030*** (0.102)	0.447*** (0.042)	1.002*** (0.101)	0.455*** (0.042)	1.003*** (0.101)
5	Electricity access (1=have electricity; 0=no electricity)	0.146 (0.201)	-0.387 (0.479)	0.105 (0.201)	-0.407 (0.479)	0.11 (0.202)	-0.345 (0.482)	0.0883 (0.201)	-0.402 (0.480)
6	Living in urban (1=urban; 0=rural)	0.101 (0.106)	-0.947*** (0.217)	0.057 (0.106)	-0.944*** (0.219)	0.093 (0.106)	-0.932*** (0.217)	0.053 (0.106)	-0.950*** (0.219)
7	Living in Java (1=Java; 0=non-Java)	-1.584*** (0.366)	-1.477*** (0.289)	-1.586*** (0.366)	-1.518*** (0.291)	-1.588*** (0.366)	-1.499*** (0.289)	-1.581*** (0.366)	-1.503*** (0.290)
8	Years of schooling	0.126*** (0.018)	0.164*** (0.031)	0.128*** (0.018)	0.173*** (0.031)	0.127*** (0.018)	0.175*** (0.031)	0.128*** (0.018)	0.169*** (0.031)
9	Age (years)	-0.048 (0.041)	0.0411*** (0.011)	-0.0455 (0.041)	0.0415*** (0.011)	-0.0461 (0.041)	0.0413*** (0.011)	-0.0468 (0.041)	0.0414*** (0.011)
	Constant	4.737*** (1.506)	-12.14*** (0.737)	4.820*** (1.507)	-12.19*** (0.755)	4.719*** (1.507)	-12.38*** (0.749)	4.915*** (1.506)	-12.14*** (0.760)
	Observations	41176	41176	41176	41176	41176	41176	41176	41176

Standard errors in parentheses.

*** p<0.01,

** p<0.05,

* p<0.1.

a IDR: one million Indonesian Rupiah about 69 US$.

Additionally, we also apply the Difference regression that excludes time invariant variables. Table 3 corroborates earlier results; it shows that individuals who received the social assistance of PKH, Rastra, KKS, or who received at least one of those programs (BANSOS), tend to have higher cigarette consumption growth compared to individuals that did not receive social assistance. Recipients of Rastra experience the largest increase in intensity, with cigarette consumption increasing by 0.402 sticks per day (2.8 sticks per week) among recipients. These findings are largely consistent with the results from the standard DiD approach, barring the significance of the PKH program. Hence, Tables 2 and 3 confirm that receiving social assistance can induce increased cigarette consumption. These findings support the previous findings in Table 1 that there exists a positive relationship between receiving social assistance and cigarette consumption, with causality between the number of cigarettes consumed by households and the massive social assistance expansion provided by the government to the poor and near-poor in Indonesia.

**Table 3:** Estimations of the impact of social assistance on cigarette consumption 2007–2014: Difference regressions

*No.*	*Variables*	*Lowest 40% SES*				*Highest 60% SES*				*All Sample*			
		*PKH*	*RASTRA*	*BLT*	*BANSOS*	*PKH*	*RASTRA*	*BLT*	*BANSOS*	*PKH*	*RASTRA*	*BLT*	*BANSOS*
		*OLS*	*OLS*	*OLS*	*OLS*	*OLS*	*OLS*	*OLS*	*OLS*	*OLS*	*OLS*	*OLS*	*OLS*
1	Receive social protection in at least one period (1=recipient; 0=never received)	0.752 (0.471)	0.841*** (0.310)	0.527** (0.223)	1.059*** (0.380)	1.579* (0.888)	0.212 (0.206)	0.696*** (0.238)	0.074 (0.208)	0.382* (0.218)	0.402*** (0.081)	0.266*** (0.079)	0.401*** (0.088)
2	Difference of per capita expenditure (in million IDR)^a^	0.714*** (0.098)	0.715*** (0.098)	0.716*** (0.098)	0.715*** (0.098)	0.315*** (0.069)	0.322*** (0.070)	0.329*** (0.070)	0.315*** (0.070)	0.437*** (0.042)	0.458*** (0.042)	0.448*** (0.042)	0.454*** (0.042)
3	Electricity access in at least one period (1=have electricity; 0=never have electricity)	0.418 (1.357)	0.364 (1.356)	0.497 (1.357)	0.442 (1.356)	-0.914 (2.589)	-1.038 (2.588)	-0.842 (2.588)	-1.056 (2.588)	1.243** (0.634)	1.269** (0.633)	1.313** (0.634)	1.293** (0.633)
4	Living in Java in at least one period (1=Java; 0=never lived in Java)	-0.709*** (0.225)	-0.780*** (0.227)	-0.712*** (0.225)	-0.749*** (0.226)	-0.814*** (0.198)	-0.838*** (0.200)	-0.821*** (0.198)	-0.814*** (0.199)	0.081 (0.077)	0.028 (0.077)	0.074 (0.077)	0.046 (0.077)
5	Living in urban area in at least one period (1=urban; 0=never lived in urban area)	0.0566 (0.224)	0.120 (0.225)	0.0569 (0.224)	0.0984 (0.224)	-0.536** (0.216)	-0.496** (0.220)	-0.511** (0.216)	-0.532** (0.218)	-0.037 (0.078)	0.052 (0.080)	-0.013 (0.078)	0.021 (0.079)
6	Difference of years of schooling	-0.013 (0.018)	-0.009 (0.018)	-0.008 (0.018)	-0.009 (0.018)	0.033** (0.016)	0.035** (0.016)	0.040** (0.016)	0.033** (0.016)	0.125*** (0.018)	0.126*** (0.018)	0.126*** (0.018)	0.126*** (0.018)
	Constant	0.007 (1.353)	-0.592 (1.374)	-0.306 (1.362)	-0.924 (1.399)	1.715 (2.584)	1.734 (2.587)	1.482 (2.585)	1.826 (2.590)	-1.102* (0.632)	-1.410** (0.635)	-1.271** (0.634)	-1.465** (0.637)
	Observations	8436	8436	8436	8436	12152	12152	12152	12152	20588	20588	20588	20588

Standard errors in parentheses.

*** p<0.01,

** p<0.05,

* p<0.1.

a IDR: one million Indonesian Rupiah about 69 US$.

Yet, as social assistance recipience itself is highly correlated with poverty, stronger conclusions require us to distinguish between the effect of poverty and cigarette consumption with that of social assistance and cigarette consumption. Errors of inclusion and exclusion in social assistance targeting allow us to conduct a robustness check by splitting our samples into two groups based on their socioeconomic status (SES): 1) the bottom 40% SES (eligible for social assistance); and 2) top 60% SES (by design, should not be eligible for social assistance but may receive it due to inclusion errors). The results show that within the two SES groups, social assistance recipients tend to have higher cigarette consumption, which is consistent with the aggregate sample results. We find that in the low 40% SES, receiving the Rastra, BLT, and at least one social assistance program (BANSOS), significantly increases the cigarette consumption of recipients compared to non-recipients. Meanwhile, the BLT and PKH significantly increase the cigarette consumption of recipients in the top 60% SES. Thus, social assistance recipients have a greater intensity of cigarette consumption compared to non-recipients, regardless of whether they come from low or high SES.

### Socioeconomic indicators: Non-smokers vs smokers

After providing evidence that social assistance strongly increases cigarette consumption, we then assessed whether there exist differences between the socioeconomic conditions of social assistance recipients who are smokers and those who are non-smokers. Table 4 shows the differences in the socioeconomic indicators of smokers and non-smokers who receive at least one kind of social assistance, with individuals divided by expenditure quintiles. A positive number implies that the indicator value for non-smokers is higher than the value for smokers; and vice-versa for negative numbers. We observe rather consistent results, where smokers have lower nutritional intake and lower health and education expenditures per capita than non-smokers. For instance, a non-smoker in the first quintile (Q1) who received at least one social assistance consumes 33.39 g per capita of protein more than a smoker who received at least one social assistance. Moreover, the results also show that smoking behavior adversely affects younger household members. We observe consistent patterns in all expenditure quintiles that children living in households with smokers generally suffer from more sick days, undergo fewer years of schooling, experience higher dropout rates, and receive less education expenditures per capita. For example, children (aged <15 years) from a non-smoker family with at least one social assistance tends to have lower inpatient days (by 0.078 days) than those from a smoker family with at least one social assistance.

**Table 4:** Comparison of socioeconomic indicators between smokers and non-smokers who are social assistance recipients (at least one social assistance)

*Indicators*	*Receiving at least one social assistance*				
	*Q1*	*Q2*	*Q31*	*Q4*	*Q51*
	*Non-smoker vs smoker*	*Non-smoker vs smoker*	*Non-smoker vs smoker*	*Non-smoker vs smoker*	*Non-smoker vs smoker*
	*Difference*	*Difference*	*Difference*	*Difference*	*Difference*
Calorie per capita (kcal/capita)	980.57***	1858.42***	2113.22***	1589.99***	727.23***
Protein per capita (g/capita)	33.39***	67.62***	74.14***	79.78***	80.97***
Fat per capita (g/capita)	35.14***	59.93***	64.16***	61.47***	43.69***
Carbohydrate per capita (g/capita)	92.92***	200.25***	242.51***	119.32***	-61.56***
Average sick days	0.348***	0.366***	0.365***	0.461***	0.389***
Average inpatient days	0.030***	0.061***	0.081***	0.188***	0.282***
Average sick days, HH member aged <15 years	-0.078***	-0.055***	-0.048**	-0.090***	-0.123***
Average inpatient days, HH member aged <15 years (days)	-0.006	-0.016*	0.016	0.011	-0.018
Years of schooling, HH members aged <15 years	0.350***	0.410***	0.402***	0.439***	0.430***
Number of HH members aged <15 years dropout (child)	-0.007***	-0.006***	-0.007***	-0.004***	-0.001**
Education expenditure per capita (IDR/capita)^a^	417.72***	477.20***	837.77***	2255.16***	11594.03***
Health expenditure per capita (IDR/capita)^a^	655.75***	1901.44***	3476.22***	9103.88***	21229.06***

HH: household.

*** p<0.01,

** p<0.05,

* p<0.1.

a IDR: one million Indonesian Rupiah about 71 US$ in 2017.

Table 5 shows more detailed differences between smokers and non-smokers by type of social assistance. In line with the objective of the program, Rastra has the greatest impact on the nutritional intake of households compared to other social assistance programs. Furthermore, we find a consistent pattern that children from a family of smokers have more sick days and higher dropout rates. Likewise, the education and health expenditures per capita in a smoker’s household are less than those of non-smokers. Such patterns are not only consistent between recipients and nonrecipients of social assistance, but are also consistent across all quintiles of household expenditures. Hence, the findings of Tables 4 and 5 provide a clear picture that smoking behavior decreases the effectiveness of social assistance programs.

**Table 5:** Comparison of socioeconomic indicators between smokers and non-smokers of each social assistance recipient

*Indicators*	*All sample*	*One of soc. ass. (at least)*	*Rastra*	*PKH*	*PIP*	*KIS*	*KKS*
	*Non-smoker vs smoker*	*Non-smoker vs smoker*	*Non-smoker vs smoker*	*Non-smoker vs smoker*	*Non-smoker vs smoker*	*Non-smoker vs smoker*	*Non-smoker vs smoker*
	*Difference*	*Difference*	*Difference*	*Difference*	*Difference*	*Difference*	*Difference*
Calorie per capita (kcal/capita)	1632.57***	1652.00***	1812.21***	740.92***	460.66***	1686.47***	1401.99***
Protein per capita (g/capita)	81.80***	72.50***	67.99***	32.79***	25.39***	75.72***	54.82***
Fat per capita (g/capita)	68.28***	60.92***	55.05***	34.45***	27.92***	64.89***	46.38***
Carbohydrate per capita (g/capita)	116.00***	144.49***	196.87***	39.84**	-5.21	141.31***	144.11***
Average sick days	0.290***	0.383***	0.472***	0.266***	0.105***	0.388***	0.483***
Average inpatient days	0.113***	0.117***	0.112***	0.070***	0.050***	0.123***	0.079***
Average sick days, HH member aged <15 years	-0.073***	-0.074***	-0.056***	-0.089***	-0.037**	-0.081***	-0.107***
Average inpatient days, HH member aged <15 years	0.010***	-0.001	-0.010	-0.036**	0.000	0.000	-0.005
Years of schooling, HH member aged <15 years	0.383***	0.380***	0.420***	0.332***	0.348***	0.348***	0.370***
Number of HH members aged <15 years dropout (child)	-0.005***	-0.005***	-0.006***	-0.008***	-0.004***	-0.006***	-0.006***
Education expenditure per capita (IDR/capita)^a^	8584.79***	2765.11***	309.21**	2749.47***	3203.58***	3369.36***	1008.66***
Health expenditure per capita (IDR/ capita)^a^	9562.63***	6732.51***	6020.17***	1520.13**	2372.80***	6857.75**	4680.54***

HH: household.

*** p<0.01,

** p<0.05,

* p<0.1.

a IDR: one million Indonesian Rupiah about 71 US$ in 2017.

## DISCUSSION

In general, our study shows that certain social assistance programs have impacts on cigarette consumption. This has been confirmed using two different datasets, each using different approaches (cross-section and panel). The impact evaluation results using the DiD method, strengthen the assertion that social assistance does have the ability to drive cigarette consumption. Using the income effect approach, increased consumption possibilities due to social assistance – either through direct cash injections or reduction of expenditures through in-kind goods/services which frees up budgets for other allocations – gives the recipient the ability to spend more on cigarettes. Thus, our findings are not consistent with the findings of Evans and Popova^17^, but are somewhat in line with those of Bazzi et al.^18^.

Evans and Popova^17^, who reviewed multiple articles around the world, found that in general, social assistance programs do not significantly affect temptation goods consumption and argue that social assistance that targeted females and strongly messaged for certain usages would lower the risk of usage for temptation goods. Bazzi et al.^18^ had found that while the initial disbursements of unconditional cash transfers do not significantly increase tobacco consumption, the second disbursement positively increased tobacco consumption. Our results regarding the PKH program corroborate the finding of Evans and Popova^17^, as the targeted nature of the PKH may contribute to the insignificance of the program’s impact on cigarette consumption in the DiD model and weak significance in the Difference regressions. Moreover, the PKH program is a conditional cash transfer, resulting in greater emphasis on usage of the assistance for health and education. However, other programs such as the Rastra and BLT that are less targeted and are unconditional have shown a tendency to increase the intensity of smoking behaviors – consistent with Bazzi et al.^18^ findings.

Although a recent working paper demonstrated that unconditional cash transfers in Indonesia lack any significant effect on smoking intention (i.e. on turning non-smokers into smokers)^19^, we note that the amount of the unconditional transfers is not large enough to induce such a drastic change. Converting non-smokers into smokers requires a greater impact than is needed to raise the intensity of existing smoking behaviors. Indeed, our findings on the rise in cigarette stick consumption complement results from Al-Izzati et al.^19^ who show that social assistance does not cause changes in smoking behavior (recipients becoming smokers). However, our findings show that social assistance causes an increase in the intensity (quantity) of smoking consumption.

In our study, the samples were split into two groups based on their SES. The first group consists of respondents in the bottom 40% SES who are eligible for social assistance, while the second group consists of respondents who are not eligible. The results show that social assistance increases the intensity (quantity) of cigarette consumption in both groups. Specifically, we also find that unconditional cash transfers (BLT) consistently drive the intensity of smoking consumption among respondents in both the bottom 40% SES and top 60% SES. This indicates that unconditional cash transfers prompt higher intensity of cigarette consumption per day. Therefore, our results show that, regardless of SES status, social assistance recipients have higher cigarette consumption compared to non-recipients. Hence, social assistance recipients having higher cigarette consumption is not a poor-specific phenomenon.

With regard to years of schooling, the cross-section method and panel method show two conflicting results. This may be due to the difference in the data utilized (as the panel result is consistent with past studies that also utilize the IFLS^26^) and the method used to calculate years of schooling (the cross-section analysis uses household average years of schooling, while the panel analysis uses the individual’s years of schooling). Additionally, previous research has in general found inconsistent results for the effect of education on smoking behaviors in Indonesia^27,26^.

Social assistance programs are meant to improve socioeconomic conditions, such as increasing schooling rates (PKH and PIP), increasing health outcomes (KIS), and increasing nutritional consumption (Rastra). However, this goal may prove difficult when social assistance drives the intensity of cigarette consumption. Results from Table 5 show that smokers have lower health and education expenditures, lower nutrition consumption, higher numbers of dropout children, and children with more sick days. This may be the result of smoking behaviors which tend to crowd-out important consumption such as nutrition^28^ and education^29^. These findings confirm other research demonstrating that smoking, which ranks as the second-largest expenditure of Indonesians, can exacerbate malnutrition^30^. When social assistance induces increased intensity of smoking behaviors, the social assistance may be rendered less effective in improving socioeconomic indicators. This may amplify the cycle of chronic poverty for social assistance recipients if smoking behaviors persist or intensify^31,32^. Concurrently, the worse socioeconomic indicators of smokers will hamper the goals of social assistance programs to raise the welfare of low-income populations.

The threat of exacerbated smoking behaviors inhibits the full potential of social assistance programs. Yet, because of the Indonesian government’s current trend in moving social assistance programs towards more targeted, integrated, and conditional programs, the possible risks of social assistance in driving cigarette consumption may be mitigated. The risks may be further mitigated if the government emphasizes the need for reduced smoking behaviors among social assistance recipients or the inclusion of conditionalities regarding smoking behaviors.

### Limitations

This study has several limitations. First, one of the biggest criticisms of the tobacco tax system in Indonesia is the multiple-tiered excise rates on different types of tobacco products. This has resulted in price gaps between tobacco products paid for by different SES groups in Indonesia (as tobacco products consumed may differ among SES). This can be proven: using the 2017 Susenas data, we are able to estimate that the average cigarette unit prices of the 10th decile is 1.9 times higher compared to the average cigarette unit prices of the 1st decile. While unit prices would be useful to use as a proxy to control for prices, it comes with a caveat that unit prices are endogenous with consumption (as the unit price is the inputted price that is derived from consumption in the dataset). Future studies should explore controlling the analysis with representative regional retail prices independent from the dataset, as price differentials may also explain the increased consumption among the poor (price effect alongside an income effect). Second, the IFLS dataset does not capture youth smoking behaviors as the questions regarding smoking behaviors are only answered by household members aged >15 years.

## CONCLUSIONS

This study has examined the persistent increase in smoking intensity in Indonesia, especially among low-expenditure populations (1st to 4th decile), which has risen faster than that in the higher deciles between 2016 and 2017. This unequal growth should present an important warning for tobacco control efforts as it implies that the low-expenditure population are increasingly able to afford tobacco products that will burden them in the future (due to long-term health risks). Crucially, our study demonstrates that recipients of social assistance programs consume more cigarettes per capita per week than non-recipients. Furthermore, our study proves that smokers will have lower socioeconomic indicators than non-smokers. Smokers tend to consume less nutrition (in terms of calories, carbohydrates, fats, and proteins) and have less education and health expenditures per capita. Moreover, we also find that smoking behavior, which is nudged and supported by the benefits of social assistance programs, can harmfully impact younger household members, resulting in them experiencing less educational attainment, higher dropout rates, and higher average sick days.

It is our hope that the findings presented here will build awareness for policymakers regarding the necessity to consider the issues that entangle social assistance programs and tobacco control efforts. Further action must be taken to compensate for the adverse impact of social assistance programs toward cigarette consumption. Therefore, we believe that it is important for policymakers to improve the distribution design of social assistance programs, inserting certain clauses to penalize smoking behaviors or reward non-smoking behaviors among social assistance recipients, especially for social assistance programs affecting the younger generation, such as the PIP and PKH. We believe that those measures will enhance the effectiveness of social assistance programs in achieving better socioeconomic impacts for those most in need.
