# Supplementary material for: Good intentions, unintended outcomes: Impact of social assistance on tobacco consumption in Indonesia
Source: Tob Induc Dis. 2021 Apr 15;19:29. doi: 10.18332/tid/132966 (PMC8049109; doi:10.18332/tid/132966)
Supplement: Supplementary file 1 [file TID-19-29-s1.pdf]

## Supplementary Table S1 – Descriptive Statistics of Susenas Dataset 2017

| Variable | Obs | Mean | Std. Dev. | Min | Max |
|---|---|---|---|---|---|
| Cigarette Consumption per Capita (Cigarette/Capita/Week) | 67,487,588 | 17 | 22 | 0 | 420 |
| Rastra Recipient (1 = Recepient; 0 = Non-Recepient) | 67,487,588 | 0.36 | 0.48 | 0 | 1 |
| PIP Recipient (1 = Recepient; 0 = Non-Recepient) | 67,487,588 | 0.10 | 0.30 | 0 | 1 |
| KKS Recipient (1 = Recepient; 0 = Non-Recepient) | 67,487,588 | 0.14 | 0.35 | 0 | 1 |
| PKH Recipient (1 = Recepient; 0 = Non-Recepient) | 67,487,588 | 0.06 | 0.24 | 0 | 1 |
| KIS Recipient (1 = Recepient; 0 = Non-Recepient) | 67,487,588 | 0.41 | 0.49 | 0 | 1 |
| At Least Receive one Social Assistance (1 = Recepient; 0 = Non-Recepient) | 67,487,588 | 0.60 | 0.49 | 0 | 1 |
| Living in Urban (1 = Urban; 0 = Rural) | 67,487,588 | 0.53 | 0.50 | 0 | 1 |
| Average Household Years of Schooling (Years) | 67,487,588 | 7 | 3 | 0 | 22 |
| 1st Expenditure Quintile (1 = 1st Quintile; 0 = Others) | 67,487,588 | 0.20 | 0.40 | 0 | 1 |
| 2nd Expenditure Quintile (1 = 2nd Quintile; 0 = Others) | 67,487,588 | 0.20 | 0.40 | 0 | 1 |
| 3rd Expenditure Quintile (1 = 3rd Quintile; 0 = Others) | 67,487,588 | 0.19 | 0.39 | 0 | 1 |
| 4th Expenditure Quintile (1 = 4th Quintile; 0 = Others) | 67,487,588 | 0.19 | 0.39 | 0 | 1 |
| 5th Expenditure Quintile (1 = 5th Quintile; 0 = Others) | 67,487,588 | 0.22 | 0.41 | 0 | 1 |
| Living in Java (1 = Java; 0 = Non-Java) | 67,487,588 | 0.59 | 0.49 | 0 | 1 |
| Living in Sumatra (1 = Java; 0 = Non-Java) | 67,487,588 | 0.21 | 0.41 | 0 | 1 |
| Living in Nusa Tenggara (1 = Java; 0 = Non-Java) | 67,487,588 | 0.05 | 0.22 | 0 | 1 |
| Living in Kalimantan (1 = Java; 0 = Non-Java) | 67,487,588 | 0.06 | 0.24 | 0 | 1 |
| Living in Sulawesi (1 = Java; 0 = Non-Java) | 67,487,588 | 0.07 | 0.25 | 0 | 1 |
| Living in Maluku-Papua (1 = Java; 0 = Non-Java) | 67,487,588 | 0.02 | 0.15 | 0 | 1 |
| Electricity Access (1 = Have Electricity; 0 = No Electricity) | 67,487,588 | 0.98 | 0.13 | 0 | 1 |
| Calorie Per Capita (Kcal/Capita/Week) | 67,487,588 | 31,528 | 9,191 | 14,003 | 62,999 |
| Protein Per Capita (Gram/Capita/Week) | 67,487,588 | 920 | 340 | 51 | 5,413 |
| Fat Per Capita (Gram/Capita/Week) | 67,487,588 | 816 | 342 | 20 | 3,730 |
| Carbohydrate Per Capita (Gram/Capita/Week) | 67,487,588 | 4,677 | 1,395 | 645 | 13,863 |
| Average Sick Days (Days) | 67,487,588 | 0.90 | 2.39 | 0 | 30 |
| Average Inpatient Length (Days) | 67,487,588 | 0.25 | 1.22 | 0 | 135 |
| Average Sick Days HH Members Aged Below 15 Years Old (Days) | 43,377,681 | 0.70 | 1.90 | 0 | 30 |
| Average Inpatient Length HH Members Aged Below 15 Years Old (Days) | 43,377,681 | 0.18 | 1.23 | 0 | 175 |
| Average Years of Schooling HH Members Aged Below 15 Years Old (Years) | 43,377,681 | 2.92 | 2.74 | 0 | 11 |
| Average Number of Child Dropouts (Children) | 67,487,588 | 0.01 | 0.10 | 0 | 4 |
| Education Per Capita (Rupiah/Capita) | 67,487,588 | 22,766 | 103,094 | 0 | 14,100,000 |
| Health Per Capita (Rupiah/Capita) | 67,487,588 | 20,641 | 116,077 | 0 | 17,900,000 |

Source: Authors' calculation

## Supplementary Table S2 – Descriptive Statistics of IFLS Dataset 2007 - 2014

| Variable | Obs | Mean | Std. Dev. | Min | Max |
|---|---|---|---|---|---|
| Average Cigarette Consumption per Day (Cigarette/Day) | 41,176 | 3.74 | 6.97 | 0 | 80 |
| PKH Recipient (1 = Recipient; 0 = Non-Recipient) | 41,176 | 0.02 | 0.12 | 0 | 1 |
| Raskin (Rastra) Recipient (1 = Recipient; 0 = Non-Recipient) | 41,176 | 0.51 | 0.50 | 0 | 1 |
| BLT Recipient (1 = Recipient; 0 = Non-Recipient) | 41,176 | 0.25 | 0.43 | 0 | 1 |
| At Least Receive one Social Assistance (1 = Recipient; 0 = Non-Recipient) | 41,176 | 0.61 | 0.49 | 0 | 1 |
| Per Capita Expenditure (in Million Rupiah) | 41,176 | 0.81 | 0.81 | 0.03 | 20.08 |
| Electricity Access (1 = Have Electricity; 0 = No Electricity) | 41,176 | 0.98 | 0.15 | 0 | 1 |
| Living in Urban (1 = Urban; 0 = Rural) | 41,176 | 0.54 | 0.50 | 0 | 1 |
| Living in Java (1 = Java; 0 = Non-Java) | 41,176 | 0.57 | 0.50 | 0 | 1 |
| Years of Schooling (Years) | 41,176 | 7.97 | 4.45 | 0 | 22 |
| Age (Years) | 41,176 | 39.53 | 14.72 | 14 | 107 |

Source: Authors' calculation

## Supplementary Table S3 – Tobit Regression of Cigarette Consumption in 2016 (stick per capita per week)

| No. | Variables | Tobit Regression | | | | |
|---|---|---|---|---|---|---|
| | | Cigarette Per Capita | Cigarette Per Capita | Cigarette Per Capita | Cigarette Per Capita | Cigarette Per Capita |
| 1 | Recipient of Rastra (1 = Recipient; 0 = Non-Recipient) | 4.983*** | | | | |
| | | (0.00892) | | | | |
| 2 | Recipient of PIP (1 = Recipient; 0 = Non-Recipient) | | 1.999*** | | | |
| | | | (0.0122) | | | |
| 3 | Recipient of KKS (1 = Recipient; 0 = Non-Recipient) | | | 3.288*** | | |
| | | | | (0.0107) | | |
| 4 | Recipient of KIS (1 = Recipient; 0 = Non-Recipient) | | | | 2.420*** | |
| | | | | | (0.00894) | |
| 5 | Recipient of At Least One Social Protection (1 = Recipient; 0 = Non-Recipient) | | | | | 5.354*** |
| | | | | | | (0.00901) |
| 6 | Urban (1 = Urban; 0 = Rural) | -4.255*** | -4.982*** | -4.929*** | -4.961*** | -4.255*** |
| | | (0.00862) | (0.00853) | (0.00853) | (0.00852) | (0.00860) |
| 7 | Average Household Member Years of Schooling | -0.550*** | -0.680*** | -0.639*** | -0.641*** | -0.527*** |
| | | (0.00153) | (0.00152) | (0.00152) | (0.00153) | (0.00154) |
| 8 | 2nd Expenditure Quintile (1 = 2nd Quintile; 0 = Others) | 8.117*** | 7.830*** | 7.968*** | 7.881*** | 8.127*** |
| | | (0.0123) | (0.0123) | (0.0123) | (0.0123) | (0.0123) |
| 9 | 3rd Expenditure Quintile (1 = 3rd Quintile; 0 = Others) | 13.01*** | 12.39*** | 12.61*** | 12.41*** | 12.98*** |
| | | (0.0126) | (0.0126) | (0.0126) | (0.0125) | (0.0126) |
| 10 | 4th Expenditure Quintile (1 = 4th Quintile; 0 = Others) | 16.59*** | 15.59*** | 15.87*** | 15.63*** | 16.61*** |
| | | (0.0131) | (0.0131) | (0.0131) | (0.0130) | (0.0131) |
| 11 | 5th Expenditure Quintile (1 = 5th Quintile; 0 = Others) | 20.50*** | 18.90*** | 19.21*** | 19.02*** | 20.76*** |
| | | (0.0143) | (0.0141) | (0.0141) | (0.0140) | (0.0144) |
| 12 | Living in Sumatera (1 = Sumatera; 0 = Others) | 5.093*** | 4.212*** | 4.304*** | 4.079*** | 4.584*** |
| | | (0.0101) | (0.0100) | (0.0100) | (0.0100) | (0.0100) |
| 13 | Living in Nusa Tenggara (1 = Nusa Tenggara; 0 = Others) | -5.971*** | -6.520*** | -6.501*** | -6.827*** | -6.795*** |
| | | (0.0182) | (0.0182) | (0.0182) | (0.0182) | (0.0182) |
| 14 | Living in Kalimantan (1 = Kalimantan; 0 = Others) | 2.179*** | 0.788*** | 0.956*** | 0.675*** | 1.575*** |
| | | (0.0170) | (0.0169) | (0.0169) | (0.0169) | (0.0169) |
| 15 | Living in Sulawesi (1 = Sulawesi; 0 = Others) | 3.053*** | 1.976*** | 2.005*** | 1.898*** | 2.507*** |
| | | (0.0160) | (0.0159) | (0.0159) | (0.0159) | (0.0159) |
| 16 | Living in Maluku-Papua (1 = Maluku-Papua; 0 = Others) | -7.684*** | -8.793*** | -8.951*** | -9.208*** | -8.902*** |
| | | (0.0273) | (0.0273) | (0.0273) | (0.0273) | (0.0272) |
| 17 | Electricity (1 = Have Electricity; 0 = Others) | 0.490*** | 0.839*** | 1.167*** | 0.978*** | 0.717*** |
| | | (0.0276) | (0.0277) | (0.0277) | (0.0276) | (0.0276) |
| | Constant | 0.324*** | 4.324*** | 3.152*** | 3.495*** | -0.769*** |
| | | (0.0298) | (0.0288) | (0.0292) | (0.0291) | (0.0301) |
| | Observations | 66,698,347 | 66,698,347 | 66,698,347 | 66,698,347 | 66,698,347 |

Standard errors in parentheses, *** $p<0.01$, ** $p<0.05$, * $p<0.1$

Source: Authors' calculation

## Supplementary Table S4 – Parallel Trend

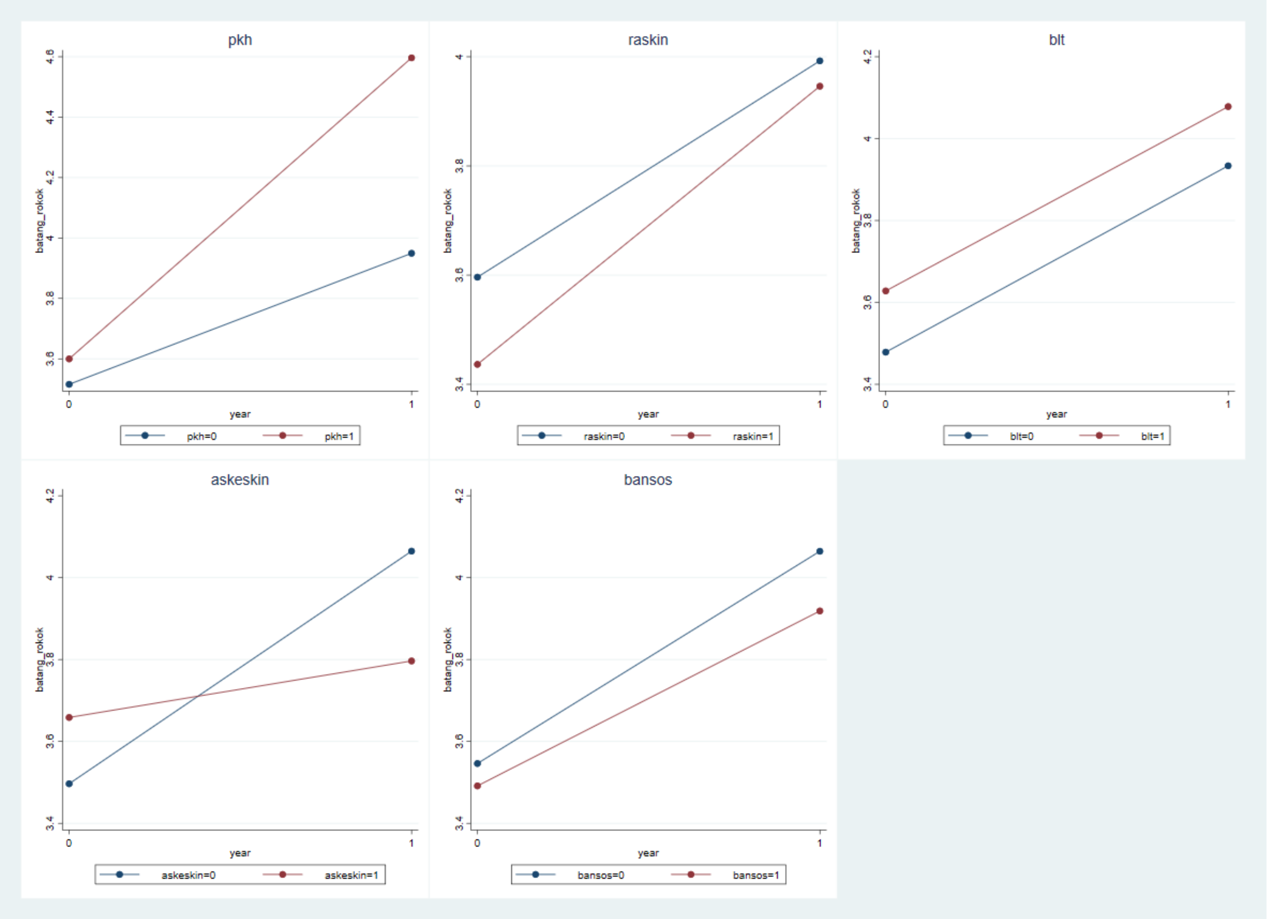


Note: year 0 = 2007 year 1 = 2014; raskin = Rastra; BLT= Cash Transfer (KKS); Bansos = at least receiving one social assistance; Askeskin = a former form of KIS; Batang Rokok = Cigarette Stick.
*Source: Authors' calculation.*

© 2021 Dartanto T. et al.
